# Inhibition of lung serine proteases in mice: a potentially new approach to control influenza infection

**DOI:** 10.1186/1743-422X-8-27

**Published:** 2011-01-20

**Authors:** Mahmoud M Bahgat, Paulina Błazejewska, Klaus Schughart

**Affiliations:** 1 Department of Infection Genetics and University of Veterinary Medicine Hannover, Helmholtz Centre for Infection Research, Braunschweig, Germany; 2 Therapeutical Chemistry Department, Immunology and Infectious Diseases Group, the Center of Excellence for Advanced Sciences, the National Research Center, Dokki, Cairo 12311, Egypt

## Abstract

**Background:**

Host serine proteases are essential for the influenza virus life cycle because the viral haemagglutinin is synthesized as a precursor which requires proteolytic maturation. Therefore, we studied the activity and expression of serine proteases in lungs from mice infected with influenza and evaluated the effect of serine protease inhibitors on virus replication both in cell culture and in infected mice.

**Results:**

Two different inbred mouse strains were investigated: DBA/2J as a highly susceptible and C57Bl/6J as a more resistant strain to influenza virus infection. The serine proteases from lung homogenates of mice exhibited pH optima of 10.00. Using the substrate Bz-Val-Gly-Arg-*p*-nitroanilide or in zymograms, the intensities of proteolysis increased in homogenates from both mouse strains with time post infection (p.i.) with the mouse-adapted influenza virus A/Puerto Rico/8/34 (H1N1; PR8). In zymograms at day 7 p.i., proteolytic bands were stronger and numerous in lung homogenates from DBA/2J than C57Bl/6J mice. Real-time PCR results confirmed differential expression of several lung proteases before and after infecting mice with the H1N1 virus. The most strongly up-regulated proteases were *Gzma*, *Tmprss4*, *Elane*, *Ctrl*, *Gzmc *and *Gzmb*. Pretreatment of mouse and human lung cell lines with the serine protease inhibitors AEBSF or *p*AB or a cocktail of both prior to infection with the H1N1 or the A/Seal/Massachusetts/1/80 (H7N7; SC35M) virus resulted in a decrease in virus replication. Pretreatment of C57Bl/6J mice with either AEBSF or a cocktail of AEBSF and *p*AB prior to infection with the H1N1 virus significantly reduced weight loss and led to a faster recovery of treated versus untreated mice while *p*AB alone exerted a very poor effect. After infection with the H7N7 virus, the most significant reduction of weight loss was obtained upon pretreatment with either the protease inhibitor cocktail or *p*AB. Furthermore, pretreatment of C57BL/6J mice with AEBSF prior to infection resulted in a significant reduction in the levels of both the H1N1 and H7N7 nucleoproteins in mice lungs and also a significant reduction in the levels of the HA transcript in the lungs of the H1N1- but not the H7N7-infected mice.

**Conclusion:**

Multiple serine protease activities might be implicated in mediating influenza infection. Blocking influenza A virus infection in cultured lung epithelia and in mice by the used serine protease inhibitors may provide an alternative approach for treatment of influenza infection.

## Background

Hemagglutinin (HA) of influenza virus is responsible for binding of virus particles to sialic acid-containing cell surface receptors. It is synthesized as a precursor protein HA0 that needs to be cleaved by a host protease(s) into HA1 and HA2 subunits to gain its fusion ability to host cell membrane and thereby initiate the infection process [1-4]. The cleavage site of HA0 of most avian and mammalian influenza viruses is monobasic and carries a single arginine, rarely a single lysine amino acid. Cleavage has been reported to occur extracellularly by trypsin [5,6], trypsin-like proteases such as plasmin [7-9], tryptase Clara from rat bronchiolar epithelial Clara cells, mast cell tryptase from porcine lung [10] and an analogous protease from chicken allantoic fluid to the blood clotting factor Xa [11] or bacterial proteases [12,13].

The transmembrane serine proteases TMPRSS2 (also known as epitheliasin) and TMPRSS11D (also known as human airway trypsin-like protease, HAT) were reported to mediate HA cleavage of A/Memphis/14/96 (H1N1), A/Mallard/Alberta/205/98 (H2N9) and A/Texas/6/96 (H3N2) [14]. Also, the involvement of the TMPRSS2 and TMPRSS4 in cleavage of the 1918 H1N1-HA was reported [15]. HAT and TMPRSS2 are synthesized as zymogens and require proteolytic cleavage at a highly conserved arginine residue to become enzymatically active and such cleavage was reported to occur autocatalytically [16,17]. The catalytic domains of the TMPRSS were thought to be only linked to the membrane-bound N-terminal chain of the enzyme by a disulfide bridge; however, soluble forms of the HAT and TMPRSS2 were also reported suggesting possible release of the catalytic domains from the cell surface [16,18]. Upon doxycycline-induced expression of HAT and TMPRSS2 in MDCK cells [19] and using both seasonal influenza virus A/Memphis/14/96 (H1N1) and pandemic virus A/Hamburg/5/2009 (H1N1), TMPRSS2 was found to cleave HA within the cell, while, HAT does it at the cell surface, thus, supporting cleavage of both newly synthesized HA and incoming virions [17]. Both activities could be blocked by appropriate peptide mimetic protease inhibitors [17].

In addition to the TMPRSS and HAT proteases that originate from lung cells, other serine proteases were reported to be expressed by infiltrating immune cells under various pro-inflammatory, inflammatory, infection and pathological circumstances [20-36]. These serine proteases might also be implicated in HA cleavage since they have the same catalytic triad present in the active site of the HAT and TMPRSS.

In the present work, the activities of trypsin-like serine proteases in lung homogenates from influenza-infected mice were characterized. In addition, the levels of transcripts encoding known serine proteases from either lungs or immune infiltrates were quantified by real-time PCR before and after infecting mice with the H1N1 subtype. Furthermore, the effects of specific serine protease inhibitors on the replication of the H1N1 and H7N7 subtypes were demonstrated both *in vitro *and *in vivo*.

## Results

### Multiple serine protease activities can be detected in lung homogenates from influenza virus-infected C57Bl/6J and DBA/2J mice

**Figure 1:**
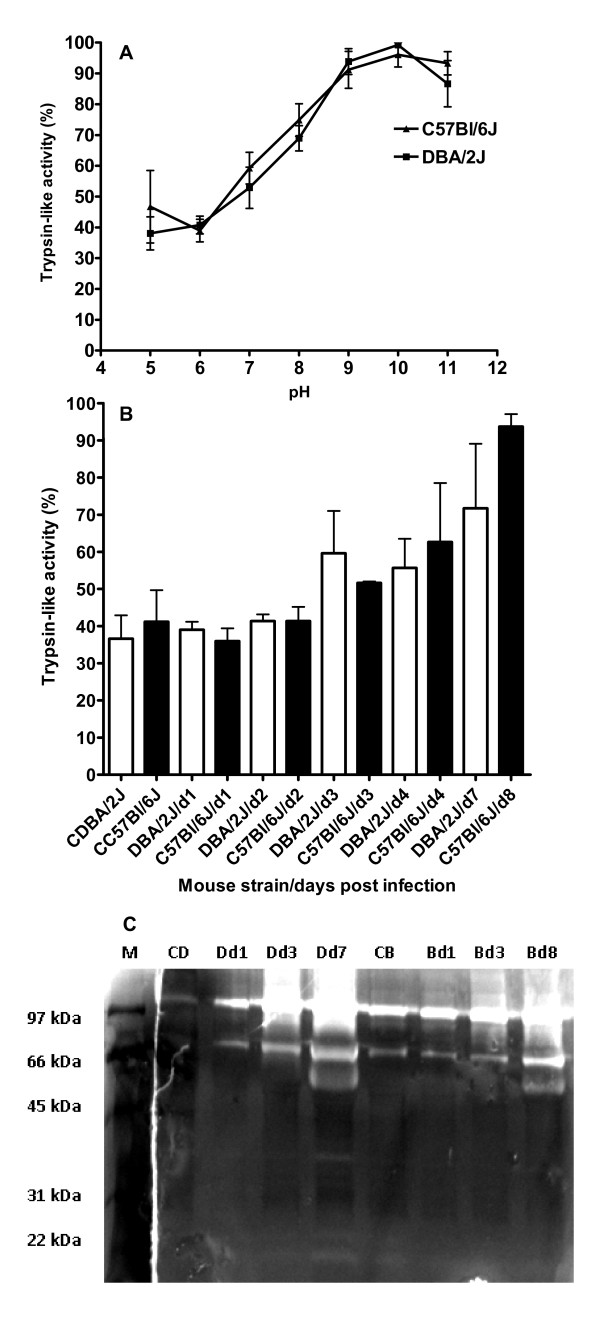
**Quantification and visualization of serine protease activities in lung homogenates from C57Bl/6J and DBA/2J**. Each mouse was infected intra-nasally with 2 × 10^3 ^FFU of the H1N1 PR8 virus and lung homogenates were prepared at different days p.i. A) The detected trypsin-like protease activity in lung homogenates from infected C57BL/6J and DBA/2J mice (pooled from day 3, 4 and 6 p.i.) using the specific substrate Bz-Val-Gly-Arg-*p*-NA had an alkaline pH optimum. Each data point represents the mean of three individual measurements (+/- 1 SD) in pooled lung homogenates from three individual mice. B) At the optimal pH 10.00, the serine protease activities (mean values +/- 1 SD) in lung homogenates from both mouse strains (n = 3 mice for each time point) showed a gradual increase with time after infection with no significant differences (P > 0.05) between lung homogenates from C57Bl/6J (black bars) and DBA/2J (white bars) mice. C) Zymograms showing the molecular weights of proteolytic enzyme activities in lung homogenates from uninfected (CD) or infected DBA/2J mice at days 1 (Dd1), 3 (Dd3) and 7 (Dd7) p.i., respectively, and uninfected (CB) or infected C57BL/6J mice at days 1 (Bd1), 3 (Bd3) and 7 (Bd7) p.i., respectively.

For the analysis of protease activities, the substrate Bz-Val-Gly-Arg-*p*-nitroanilide (*p-*NA) was used which favors cleavage by trypsin-like serine proteases. Homogenates from lungs of uninfected and PR8 (H1N1)-infected C57Bl/6J and DBA/2J mice revealed protease activities with an optimum pH of 10.00 (Figure 1A). These serine protease activities showed a gradual increase with time after infection with PR8 but no significant differences between the two mouse strains were noted (Figure 1B). In zymograms (Figure 1C) which were developed at the optimum of pH 10.00, serine protease activities in lung homogenates from both strains showed a gradual increase with time p.i. At day 1, two enzymatically active peptides were observed at molecular weights (MW) of about 97 & 66 kDa, and the intensities of these bands markedly increased at day 3 p.i. in lung homogenates from both mouse strains compared to uninfected controls. However, the proteolytic activities were in general stronger in DBA/2J than C57Bl/6J mice. At day 7 p.i., an additional proteolytic band at MW of about 56 kDa was detected in both mice strains and the intensity of all bands was stronger in DBA/2J compared to C57BL/6J mice. Also, lung homogenates from DBA/2J mice showed three additional faint activities at MW of 16, 24 and 38 kDa that were not evidenced in C57Bl/6J.

**Figure 2:**
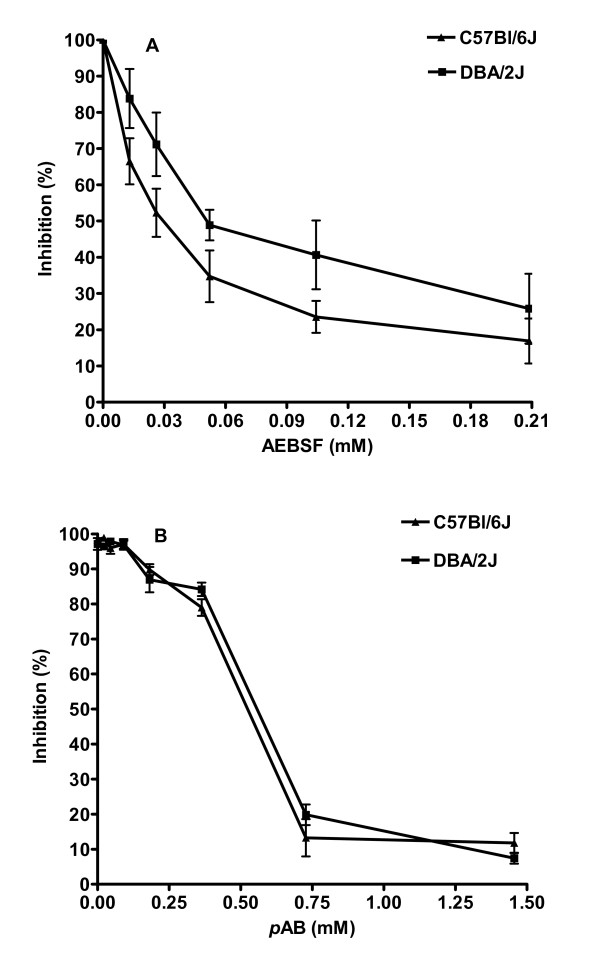
**Specific inhibitors confirm the serine protease nature of measured activity**. Each mouse was infected intra-nasally with 2 × 10^3 ^FFU of the H1N1 PR8 virus and lung homogenates were prepared at different days p.i. Serial dilutions of the inhibitors were added to extracts (pooled from day 3, 4 and 6 p.i.) prior to incubation with the substrate and the protease activities were determined. The results are presented as percent inhibition with reference to activities in untreated extracts. AEBSF (A) and *p*AB (B) reduced the protease activities in a concentration-dependent manner. Each data point represents the mean of four individual measurements +/- 1 SD.

The quantified serine protease activities from lung homogenates of both mouse strains could be inhibited by the serine protease-specific inhibitors AEBSF and *p*AB in a concentration dependent pattern (Figures 2A, B). The IC50 values for AEBSF and *p*AB for C57Bl/6J lung extracts were 0.0327 and 0.536 mM, respectively, whereas for DBA/2J lung extracts the IC50 values were 0.053 and 0.582 mM, respectively.

### Influenza infection is associated with expression of several serine protease transcripts in mouse lungs

Relative quantification of transcripts of known serine protease genes in the transcriptome of C57Bl/6J or DBA/2J infected mouse lungs revealed that the most strongly expressed proteases were *Gzmb *(*granzyme B*; only in C57Bl/6J at day 6 p.i.), *Gzma *(*granzyme A*), *Tmprss4*, *Gzmc *(*granzyme C*; only at days 1,3 p.i.), *Elane *(*neutrophil elastase*) and *Ctrl *(*chymotrypsin-like*; Table 1). The levels of transcripts encoding other proteases were much less abundant (Table 1).

**Table 1:** Expression profiles of transcripts encoding lung proteases at various times after influenza infection of C57BL/6J and DBA/2J mice with PR8 virus

Gene				**Relative quantification (2**^**-dct**^**)/Days post infection**								
	Day 0			Day 1			Day 3			Day 6		
	B6	DBA	P-value	B6	DBA	P-value	B6	DBA	P-value	B6	DBA	*P-value*
*Ctsd*	0.014 ± 0.0023	0.0054 ± 1.260E-06	**<0.05**	0.0077 ± 0.00018	0.0068 ± 0.0005	**>0.05**	0.0042 ± 0.00039	0.0082 ± 0.00028	**<0.05**	0.0027 ± 0.00046	0.0016 ± 2.192E-08	**<0.05**
*Ctrl*	0.1079 ± 0.0275	0.1705 ± 0.0581	**>0.05**	0.1031 ± 0.0179	0.1359 ± 0.0166	**>0.05**	0.1353 ± 0.054	0.1403 ± 0.03	**>0.05**	0.1018 ± 0.0046	0.1029 ± 0.0161	**>0.05**
*Gzma*	0.989 ± 0.0042	0.884 ± 0.012	**<0.05**	0.866 ± 0.0271	0.973 ± 0.0094	**<0.05**	0.9523 ± 0.0213	0.9874 ± 0.0071	**>0.05**	0.9941 ± 0.0016	0.9537 ± 0.0367	**>0.05**
*Gzmb*	0.0108 ± 0.0053	0.0390 ± 0.0139	**<0.05**	0.0095 ± 0.0041	0.0152 ± 0.0062	**>0.05**	0.0129 ± 0.0036	0.0085 ± 0.0023	**>0.05**	4.761 ± 0.0034	0.0285 ± 1.671	**<0.05**
*Gzmc*	0.0134 ± 0.0004	0.0059 ± 0.0042	**>0.05**	0.004 ± 0.0002	0.2327 ± 0.0148	**<0.05**	0.004 ± 0.0012	0.258 ± 0.0114	**<0.05**	0.108 ± 0.0393	0.0285 ± 0.0034	**<0.05**
*Gzmg*	0.0066 ± 0.0007	0.0017 ± 8.538E-06	**<0.05**	0.0017 ± 0.0009	0.0044 ± 0.0012	**>0.05**	0.0036 ± 0.0002	0.0024 ± 0.0003	**<0.05**	0.0052 ± 0.0016	0.0027 ± 0.0003	**>0.05**
*Gzmk*	0.0079 ± 0.0021	0.0042 ± 0.0011	**>0.05**	0.0036 ± 0.0006	0.0026 ± 0.0003	**>0.05**	0.004 ± 0.00043	0.003 ± 0.00018	**<0.05**	0.0097 ± 0.0009	0.0062 ± 0.0004	**<0.05**
*Mmp 1a*	0.0294 ± 0.0047	0.0057 ± 8.380E-05	**<0.05**	0.01165 ± 0.0025	0.0067 ± 0.0029	**>0.05**	0.0061 ± 0.0004	0.0084 ± 0.0016	**>0.05**	0.0062 ± 0.0021	0.0197 ± 0.0044	**<0.05**
*Mmp 1b*	0.0097 ± 0.0007	0.0056 ± 0.0002	**<0.05**	0.0071 ± 0.0011	0.0055 ± 8.095E-05	**>0.05**	0.0061 ± 0.0005	0.0035 ± 0.0007	**<0.05**	0.005 ± 0.0004	0.003 ± 0.0008	**<0.05**
*Mmp 2*	0.00012 ± 7.071E-06	0.0002 ± 9.441E-05	**>0.05**	0.00016 ± 4.543E-05	0.00019 ± 1.790E-05	**>0.05**	7.7 E-05 ± 1.90E-05	0.00011 ± 1.579E-06	**<0.05**	0.00016 ± 3.126E-05	0.00011 ± 1.331E-05	**>0.05**
*Mmp 8*	0.00055 ± 0.00026	0.00021 ± 3.525E-05	**>0.05**	0.0003 ± 4.16E-05	0.00038 ± 0.00018	**>0.05**	0.00046 ± 0.0002	0.00176 ± 6.903E-05	**<0.05**	0.00122 ± 0.0005	0.00124 ± 0.0009	**>0.05**
*Mmp 9*	0.00116 ± 0.0002	0.00036 ± 0.0004	**<0.05**	0.003 ± 8.889E-05	0.0013 ± 0.0001	**<0.05**	0.00043 ± 0.00015	0.00172 ± 3.380E-05	**<0.05**	0.0006 ± 0.0007	0.0008 ± 0.0005	**>0.05**
*Elane*	0.1555 ± 0.0198	0.2328 ± 0.0349	**<0.05**	0.2083 ± 0.023	0.1490 ± 0.040	**>0.05**	0.3728 ± 0.0820	0.2061 ± 0.039	**<0.05**	0.1838 ± 0.015	0.1706 ± 0.034	**>0.05**
*Tmprss2*	0.02647 ± 0.0181	0.03916 ± 0.0344	**>0.05**	0.00682 ± 0.01076	0.03398 ± 0.0273	**>0.05**	0.00305 ± 0.00343	0.02584 ± 0.03484	**>0.05**	0.00521 ± 0.00066	0.00232 ± 0.0017	**>0.05**
*Tmprss4*	0.2649 ± 0.1166	0.4376 ± 0.1963	**>0.05**	0.1990 ± 0.2406	0.7521 ± 0.3214	**>0.05**	0.2988 ± 0.08959	0.3265 ± 0.1309	**>0.05**	0.4279 ± 0.0147	0.03122 ± 0.0139	**<0.05**
*Tpsg1*	0.1033 ± 0.0492	0.06162 ± 0.0117	**>0.05**	0.04691 ± 0.05	0.0441 ± 0. 0.05	**>0.05**	0.1455 ± 0.0917	0.05375 ± 0.0187	**>0.05**	0.00418 ± 0.0017	0.0281 ± 0.0089	**<0.05**

Each relative quantification value represents the mean of three independent measurements using RNA from three individual C57Bl/6J or DBA/2J mice at the indicated time points post infection with the A/Puerto Rico/8/34 (H1N1; PR8) influenza virus. The difference in the levels of expression of various protease genes among the 2 mouse strains was considered significant when P-value was < 0.05.

The expression levels of *Tmprss2 *were generally low in both mouse strains before infection and at days 1 to 6 p.i., with slight but not significantly higher levels in lungs from DBA/2J mice until day 3 p.i. In both mouse strains, the levels of the *Tmprss4 *gene were significantly higher than *Tmprss2 *(P < 0.05). Whereas the level of *Tmprss4 *transcripts was not significantly higher in DBA/2J compared to C57Bl/6J mice before infection (1.7 fold) and at day 1 p.i. (3.7 fold), comparable levels were recorded in both mouse strains at day 3 p.i. After day 3, the transcript level was significantly up-regulated in C57Bl/6J compared to DBA/2J. The levels of the *Tpsg1 *(*tryptase gamma 1*) transcript before infection and at day 3 p.i. were not significantly higher in C57Bl/6J (1.6 and 2.8 fold respectively) compared to DBA/2J mice, whereas at day 6 p.i., the levels were significantly higher in C57Bl/6J compared to DBA/2J mice.

Before infection the levels of *Gzma *transcripts were significantly higher in C57Bl/6J compared to DBA/2J mice, at day 1 p.i. levels were significantly lower and thereafter similar expression levels were found in both strains. Expression of *Gzmb *transcript was significantly higher in DBA/2J than C57Bl/6J mice before infection, at days 1 and 2 p.i. It was gradually down-regulated in DBA/2J mice but slightly increased in C57Bl/6J, and became strongly up-regulated compared to all other proteases at day 6 p.i. The levels of *Gzmc *transcripts were significantly higher in DBA/2J than C57Bl/6J mice at days 1 and 3 p.i. whereas the opposite was observed at day 6 p.i. The levels of the *Gzmg *(*granzyme G*) transcript were significantly higher in C57Bl/6J mice before infection and at days 3 and 6 p.i. The same observation was made for *Gzmk *at days 3 and 6 p.i.

Prior to infection, and at day 1 p.i. the expression levels of the *Mmp1a *(*matrix metallopeptidase 1a*) gene were significantly higher in C57Bl/6J than DBA/2J mice. This situation was reversed at day 6 p.i. *Mmp1b *(*matrix metallopeptidase 1b*) transcripts showed a higher level in C57Bl/6J than DBA/2J mice and the difference was significant before, at days 3 and 6 p.i. The levels of the *Mmp2 *(*matrix metallopeptidase 2*) transcript were least expressed in both mouse strains compared to other *Mmp *genes. *Mmp8 *(*matrix metallopeptidase 8*) transcript levels were significantly higher DBA/2J than C57Bl/6J at day 3 p.i. Prior to infection and shortly thereafter the *Mmp9 *(*matrix metallopeptidase 9) *expression levels were significantly up-regulated in C57Bl/6J compared to DBA/2J mice and the opposite was recorded at day 3 p.i.

No significant differences were observed for the levels of the *Ctrl *transcript between the two mouse strains. Prior to infection, the levels of the *Elane *transcripts were significantly higher in DBA/2J mice, were somewhat higher (1.3 fold) in C57Bl/6J at day 1 p.i and became significantly higher at day 3 p.i. and comparable levels were observed in both mouse strains at day 6 p.i. While the levels of the *Ctsd *(*cathepsin D*; also known as *aspartyl proteinase*) transcripts were significantly higher in C57Bl/6J prior to infection and at day 6 p.i., comparable transcript levels were recorded in the lungs of both strains at day 1 p.i. whereas at day 3 p.i. transcript levels were higher in DBA/2J mice.

### Serine protease inhibitors block influenza A viruses propagation in cultured lung cell lines

**Figure 3:**
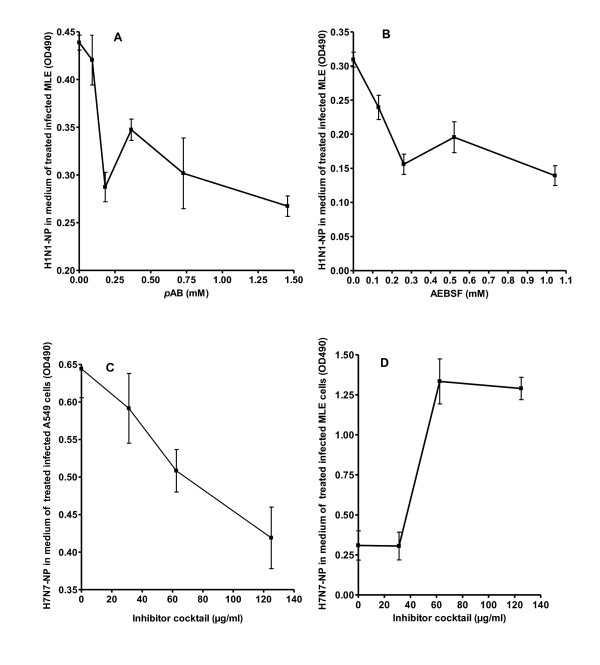
**Addition of protease inhibitors reduced influenza virus propagation in mouse and human lung cell cultures**. Mouse (MLE15) or human (A549) cell lines were infected with the PR8 virus at a multiplicity of infection of 0.01 and the amount of the virus NP in the supernatant was determined by NP-specific ELISA. Pretreatment of cultured MLE15 cells with serial dilutions of the serine protease inhibitors AEBSF (A; 0.13-1 mM) or *p*AB (B; 1.5-0.09 mM) prior to infection resulted in a decrease of the released virus particles as measured by a decrease in the amount of the viral NP in the supernatants at 24 hours p.i. (n = 3 cell culture wells for each inhibitor concentration). The lowest NP levels were recorded at the highest inhibitor concentration. A similar effect was observed upon pretreatment of A549 (n = 3 cell culture wells at each concentration) with serial concentrations of a cocktail consisting of AEBSF and *p*AB (C; 125-31 μg/ml of both inhibitors) prior to infection with H7N7 virus at a MOI 0.01. Pretreatment of MLE15 cells (n = 3 cell culture wells at each concentration) with the serine protease inhibitor cocktail (125-31 μg/ml of both inhibitors) followed by incubation of cells with H7N7 for 1 hour and then collection of medium (D) revealed that wells treated with higher cocktail inhibitor concentrations had higher NP titers in the supernatant than wells treated with lower concentrations indicating inhibition of virus entry. Each data point represents the mean of duplicate measurements of the virus NP titer in 3 individual culture wells +/- 1 SD.

Pretreatment of MLE15 cells with serial dilutions of the serine protease inhibitors AEBSF (Figure 3A) or *p*AB (Figure 3B) prior to infection with H1N1 resulted in a significant, concentration-dependent, decrease in the levels of the virus nucleoprotein (NP) in supernatants from treated cells compared to non-treated infected cells at 24 hour p.i. These results indicate a drop in virus entry and/or replication. Since individual inhibitors showed efficacy to block H1N1 infection in MLE cells serial dilutions of a cocktail of both *p*AB and AEBSF was used to interfere with H7N7 infection. Treatment of human A549 cells with increasing concentrations of the AEBSF and *p*AB cocktail prior to infection with H7N7 (Figure 3C) also showed an inhibitory effect on the virus NP production. These results showed that virus reproduction could be also inhibited in human cells lines by the used serine protease inhibitors.

At 1 hour p.i. with the H7N7 virus, higher NP levels were measured in the supernatant of the MLE15 cells pretreated with high concentrations of the AEBSF and *p*AB cocktail (Figure 3D) compared to cells treated with low concentrations. This observation suggests that the used serine inhibitors may block the processing of HA protein which is required for binding to the cellular receptors and thus more viral particles can be found in the supernatants. Alternatively, the inhibitors might have additional unknown anti-influenza effects that are independent of the HA cleavage.

### Treatment of C57Bl/6J mice with serine protease inhibitors reduced weight loss and viral load

**Figure 4:**
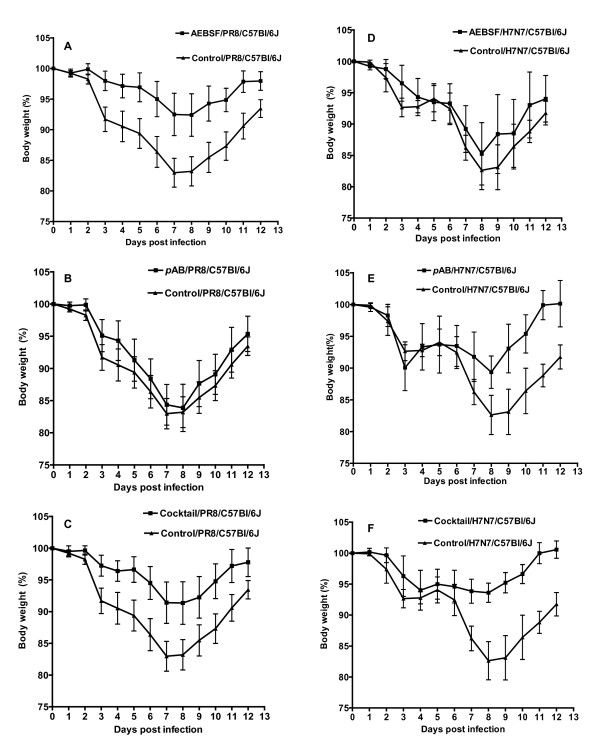
**Pretreatment of mice with serine protease inhibitors results in less severe weight loss after influenza infection**. C57BL/6J mice were pre-treated with protease inhibitors and then infected intra-nasally with 2 × 10^3 ^FFU of the H1N1 virus each. Body weight was measured at each day p.i. and is presented as percent of original weight before infection (day 0). Pretreatment of C57Bl/6J mice with AEBSF (A; 125 μg/25 μl/mouse) or with the serine protease inhibitor cocktail (C; 125 μg AEBSF, 400 μg *p*AB/25 μl/mouse) prior to infection with H1N1 (n = 8 each group) resulted in a significant reduction (P < 0.05) in the weight loss and faster recovery of treated mice compared to untreated infected controls (n = 10). On the other hand, pretreatment of C57Bl/6J mice with 400 μg *p*AB/25 μl/mouse (B; n = 8) resulted in a non significant reduction in the weight loss of treated mice compared to untreated infected controls (n = 10). AEBSF showed the lowest effect in terms of reduction of weight loss after pre-treatment of C57Bl/6J mice (n = 6) infected with H7N7 virus (D). Treatment of C57Bl/6J mice with the *p*AB (E; n = 6) or with the serine protease inhibitor cocktail (F; n = 6) at the doses described above prior to infection with the H7N7 virus resulted in a significantly (P < 0.05) reduced weight loss early after infection and a faster recovery of treated mice compared to untreated control groups (n = 6). The effect of weight loss reduction in mice treated with the inhibitor cocktail was even more pronounced after infection with the H7N7 virus compared to infection with the H1N1 virus. Each data point represents the mean percent body weight value of the tested mice +/- 1 SD.

Pretreatment of C57Bl/6J mice with AEBSF (125 μg/25 μl/mouse) prior to infection with H1N1 virus resulted in a less severe weight loss early after infection and a faster recovery of treated mice compared to untreated control groups (Figure 4A). A similar effect was obtained upon pretreatment of C57Bl/6J mice with the serine protease inhibitor cocktail (Figure 4C; 125 μg AEBSF, 400 μg *p*AB/25 μl/mouse) prior to infection with the H1N1 virus. Although pretreatment of C57Bl/6J mice with 400 μg *p*AB/25 μl/mouse (Figure 4B) resulted in a slightly faster recovery, the effect was very poor compared to that obtained by AEBSF alone or with the protease inhibitor cocktail. In contrary to its effectiveness against PR8, AEBSF showed the lowest effect in terms of weight loss reduction in C57Bl/6J mice infected with the H7N7 virus (Figure 4D). However, pretreatment of H7N7-infected C57Bl/6J mice with *p*AB or with the serine protease inhibitor cocktail (Figure 4E &4F respectively) resulted in a less severe weight loss early after infection and a faster recovery of treated mice compared to untreated control groups. Noteworthy, the weight recovery obtained upon treating mice the inhibitor cocktail prior to H7N7 infection was more prominent compared to its effect in case of H1N1 infection.

**Figure 5:**
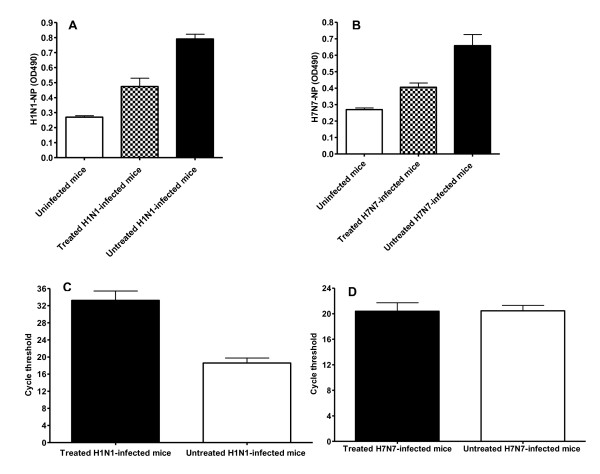
**Pretreatment with serine protease inhibitors reduced viral propagation in infected C57Bl/6J mice**. C57BL/6J mice were pre-treated with the AEBSF and then infected intra-nasally with 2 × 10^3 ^FFU of either the H1N1 or the H7N7 virus. Propagation of the two viruses in the lungs was measured by determining virus NP by ELISA and HA mRNA by real-time PCR. Uninfected mice were used as controls for the NP background signal (A, B). Pretreatment of C57Bl/6J mice with AEBSF prior to infection with H1N1 (A; n = 3) or H7N7 (B; n = 3) showed significant reduction (P < 0.05) in the levels of viral antigen at day 6 p.i compared to untreated infected mice (n = 3). All measurements were carried out in triplicates for two successive measurements on two independent days. Analysis of RNA extracted from lungs of C57Bl/6J mice that were treated with AEBSF prior to infection with H1N1 (C) revealed a significant drop in the levels of viral HA1 transcript by real-time PCR compared to untreated infected mice (n = 3 for each group). However, no significant differences in the levels of the HA transcripts were observed between the AEBSF-treated and the untreated H7N7-infected mice (D; n = 3 for each group). All real-time PCR measurements were carried out in triplicates in one experiment and the cycle threshold values of the triplicate measurements where similar. Mean values +/- 1 SD are represented.

The quantification of virus NP in lung homogenates showed that virus reproduction decreased significantly in the treated groups compared to untreated control groups, both after H1N1 (Figure 5A) and H7N7 (Figure 5B) virus infections. Furthermore, treating C57Bl/6J mice with AEBSF prior to infection with the H1N1 virus caused a significant drop in the levels of the H1N1-HA transcript compared to the untreated H1N1-infected mice (Figure 5C). However, no difference was observed in the levels of the H7N7-HA transcript between the AEBSF-treated H7N7-infected C57Bl/6J mice and the untreated H7N7-infected mice (Figure 5D) that might explain the poor effect obtained by AEBSF in terms of weight loss reduction in treated H7N7-infected C57Bl/6J mice (Figure 4D).

## Discussion

There is an urgent need for new anti-viral drugs to treat influenza infections. Therefore, we characterized protease activities in the lungs of influenza A infected mice and evaluated the effect of different protease inhibitors to viral replication *in vitro *and *in vivo*. We showed that protease activities could be detected in mouse lungs and that many protease genes are expressed before and after infection.

The protease activities in extracts from mouse lungs were studied by using the substrate Bz-Val-Gly-Arg-*p*-NA. This substrate contains an alkaline amino acid (arginine) in the P1 site upstream the PNA group that mimics the alkaline residue(s) present in the cleavage sites of the influenza A viruses HA protein and it also favors cleavage by trypsin like proteases [5-13]. The substrate cleavage assay and the zymograms gels showed that multiple protease proteins were active in mouse lungs of non-infected and infected C57Bl/6J and DBA/2J mice. These activities increased during the course of a virus infection. In infected DBA/2J mice, higher levels of activities and more proteases could be detected which may explain, in part, the higher susceptibility of DBA/2J to mouse-adapted PR8 virus and to the highly pathogenic H5N1 virus [37,38].

The involvement of numerous proteases in the process of influenza infection was further confirmed by quantifying the transcripts of known proteases in the lung tissue. The most strongly expressed proteases were *Gzmb *(only in C57Bl/6J at day 6 p.i.), *Gzma*, *Tmprss4*, *Gzmc *(only at days 1, 3 p.i.), *Elane *and *Ctrl*. Whether these proteases are directly involved in HA cleavage or may be indirectly involved in activating zymogen(s) (pre- or pro-enzymes) that are supporting HA cleavage will require further studies. The significantly higher level of transcription of *Tmprss4 *compared to *Tmprss2 *in both mouse strains suggest that this protease might play a major role in HA activation unlike its recently reported secondary role by others [39]. The best way to show which proteases are major players in influenza infection will be to study susceptibility in knock out mice that are deficient for individual protease genes. We are currently planning such experiments.

Furthermore, our results demonstrated the potential of two specific serine protease inhibitors, AEBSF [40] and *p*AB [41] or a cocktail of both to block influenza A viral replication both *in vitro *and *in vivo*. Although the function of serine protease inhibitors used in the present work are new with respect to inhibition of influenza virus replication and pathology, the approach of treating influenza infections by enzyme inhibitors adds to the observations already reported by others. Treatment of mice with the protease inhibitors epsilon-aminocaproic acid or aprotinin resulted in a faster clearance of both A/PR/8/34 (H0N1) and A/Aichi/2/68 (H3N2) in the lungs, and also non-infectious virions with uncleaved HA proteins were detected [42]. Administration of protease inhibitors gordox, contrycal and epsilon-aminocapronic acid in animal experiments or in treatments of children suffering from influenza exerted a marked antiviral and therapeutic effects. Virus particles in the lungs decreased in less pathological lesions were found [43]. Administration of the aerosolized proteinase inhibitor aprotinin by inhalation to influenza infected mice for 30-40 min incubations per day (6 micrograms/mouse/day) for 6 days allowed rescuing more than 50% of mice infected with lethal doses [44]. The serine protease inhibitor camostat was also effective in ameliorating influenza A/Taiwan/1/86 virus pathology in mice and had strong *in vitro *anti-influenza effects against amantadine-resistant type A and type B viruses [45].

Both AEBSF and *p*AB are expected to block the activity of many proteases and it remains to be seen if the effect on virus replication is restricted to the previously reported proteases *Tmprss2*, *Tmprss4 *and HAT which were shown to be directly involved in HA cleavage [15-17] or whether other proteases are also involved.

It is also conceivable that the use of protease inhibitors may exert additional indirect beneficial effects by suppressing proteases that are released from infiltrating immune cells. Such an inhibitory activity may suppress a hyper-inflammatory response in severely influenza infected individuals which has been described to be detrimental in humans and in animals. In this regard, direct neutrophil depletion using specific monoclonal antibodies increased the susceptibility of mice to infections with various influenza viruses [46-48]. In contrast, in mice infected with either the reconstructed virulent 1918 Spanish influenza pandemic H1N1 or highly pathogenic H5N1 viruses, neutrophils and macrophages predominated in the airways early after infection [49,50]. Therapeutic blockade of the neutrophil-attracting chemokine MIP-2 was associated with reduced neutrophil recruitment and a milder lung pathology following infection with mouse-adapted A/PR/8/34 virus (PR8, H1N1), suggesting that dysregulated or excessive neutrophil responses might contribute to disease during severe influenza infection [51]. The mRNA and protein expression of the IL-1 receptor-associated kinase-M (IRAK-M), an inhibitor of MyD88-dependent TLR signaling, was upregulated within 2 days after intranasal administration of PR8 [52]. The infection of IRAK-M(-/-) knock out mice resulted in substantially increased mortality compared with infected wild-type. The increased mortality was associated with enhanced early influx of neutrophils, high permeability edema, apoptosis of lung epithelial cells, markedly increased expression of inflammatory cytokines/chemokines, and release of neutrophil-derived enzymes, including myeloperoxidase and neutrophil elastase and with significantly higher viral titers in lungs and blood [52]. These results indicated that IRAK-M is critical to prevent deleterious neutrophil-dependent lung injury during influenza infection of the respiratory tract.

In inflammatory lung diseases including asthma, emphysema and chronic bronchitis, serine proteases, including the *Mmp8*, *9 *[29]*Elane*, *cathepsin G *[22] were reported to interact with structural proteins of lung cells leading to the release of neutrophil chemo-attractants which result in the recruitment of neutrophils to the site of inflammation. These effects could be reverted using specific serine protease inhibitors. The proteases involved in these processes are structurally related and share the conserved catalytic triad, His57-Asp102-Ser195 known for all serine proteases [23]. This activity can be suppressed by the inhibitors which were used in the present work.

*Elane *has a potent catalytic activity to hydrolyze elastin which ensures elasticity of the lung tissue and proteolytic resistance. Under physiological conditions, organs are protected from this enzyme by endogenous inhibitors, such as α1-protease inhibitor, α2-macrogloblin and secretory leukocyte protease inhibitor. However, in the course of a pathological condition, such as acute lung injury (ALI), the balance between *Elane *and its endogenous inhibitors is disturbed in favor of the catalytic enzyme [24-26] leading to massive infiltration of neutrophils into the lungs and subsequent tissue injury. Thus, several *Elane *inhibitors, including peptidic and nonpeptidic compounds, were used for treating ALI associated with systemic inflammation [27,28].

Granzymes are a family of conserved serine proteases stored within the cytotoxic granules of cytotoxic T-lymphocytes (CTL) [30]. There are five granzymes expressed in humans (A, B, H, K, and M) and 11 in mice (A, B, C, D, E, F, G, K, L, M, and N) [31]. *Gzmb*, perforin mRNA, CD_4_^+ ^and CD_8_^+ ^T cells levels are elevated in the BAL fluid of patients with acute respiratory inflammations mediating apoptosis of alveolar epithelial cells and leading to disease progression [32,33]. Specific inhibitors (in humans; protease inhibitor 9 and in mice protease inhibitor 6) regulate the *Gzmb *activity and minimize the enzyme-mediated apoptosis [34,35]. Influenza-specific CTL expressing both *Gzma *and *Gzmb *were reported to be dominant at early time points p.i. in the infected respiratory tract, while, at later time points, cells expressing only *Gzmb *represented the major T cell population [36].

The treatment of C57Bl/6J mice with AEBSF prior to infection with the H1N1 virus resulted in a significant decrease both in the viral NP production and HA1 transcript levels suggesting that the reduction in the weight loss was accompanied by significant drop in the viral load. Although a significant decrease in the H7N7-NP expression was achieved upon treating mice with AEBSF, the level of the HA7 transcript remained comparable to non-treated H7N7-infected mice that might explain the poor effect obtained by AEBSF in terms of weight loss reduction in treated H7N7-infected C57Bl/6J mice.

Noteworthy, the SC35M virus used in the present work is a mouse adapted H7N7 strain that was derived from the SC35. The later is a highly pathogenic H7N7 that was derived from the A_Seal_Massachussetts_1_80 H7N7 by serial passages in chicken embryo cells, thereby acquiring a multibasic (-RRRR-) HA7 cleavage site [53] that is known to be cleaved by the subtilisin-related furin [54,55] and became 100% lethal for chickens. The SC35 was then passaged 11 times in mouse lung yielding the mouse-adapted variant SC35M [56] that carries a multibasic HA cleavage site that makes SC35M more prone to cleavage by ubiquitous proteases than the monobasic cleavage site of the PR8 virus. This might be one of the reasons why HA7 transcript remained high in the AEBSF treated mice. Another possible explanation could be that the activity of the subtilisin-related furin is efficiently abolished with polybasic peptide inhibitors fused to cholromethylketone but only partially inhibited by the AEBSF inhibitor (45%; [57,58]). Thus, it has to be taken in consideration that the efficacy of the protease inhibitors to block infection might vary among various influenza subtypes depending on the susceptibility of their HAs to be cleaved by host proteases based on their cleavage site.

In contrast to SC35, which is low-pathogenic for mice, SC35M is highly pathogenic for both mice and chickens. SC35M and SC35 therefore provide a suitable system to elucidate the molecular basis of host change and enhanced virulence in mammals. SC35 and SC35M differ mainly by mutations in the polymerase proteins (PB2, PB1, and PA) and in the NP. SC35M has a considerably higher polymerase activity in mammalian cells than SC35 [59] and this could be another possible reason for the continuously high level of the HA7-RNA even after treatment. Independent of their protease inhibitory effect, the drop in the NP level although no difference in the quantified HA7-transcript might suggest an additional inhibitory effect of AEBSF on the translation of viral RNA into protein and/or assembly of the viral NP.

It is well known that proteases play crucial roles in various host functions including metabolic, protein processing, blood clotting, complement activation and immune cell recruiting activities. Therefore, before the clinical application of such a potential therapeutic approach can be envisaged, more studies on the potential toxicity and unwanted side effects will be necessary. It is, however, noteworthy that protease inhibitors are being used in different clinical settings. For example, the antiretroviral aspartyl protease inhibitors combination lopinavir/ritonavir was approved for humans. Low rate of virological failure and maintenance of susceptibility to lopinavir/ritonavir treatment were reported in clinical practice [60]. Another serine protease inhibitor that is used in humans is telaprevir [61] that specifically targets the HCV-NS3/4a serine protease.

## Conclusion

Multiple lung serine proteases might be implicated in mediating influenza infection in mice as demonstrated by both the zymography and real time PCR results. Blocking influenza A virus infection in cultured lung epithelia and in mice by serine protease inhibitors provides a potential novel approach for treatment of influenza infection.

## Methods

### Viruses, mouse strains and infection

Mouse-adapted influenza strains, A/Puerto Rico/8/34 (H1N1; PR8) and A/Seal/Massachusetts/1/80 (H7N7; SC35M), were propagated in the chorio-allantoic cavity of 10-day-old embryonated hen eggs for 48 hours at 37°C. Inbred mouse strains C57BL/6J and DBA/2J were obtained from Janvier, France. Mice were maintained under specific pathogen free conditions and all experiments were approved by an external committee according to the German regulations and laws on animal welfare. For infection experiments, mice were anesthetized by intra-peritoneal injection with Ketamin (Bayer Health Care; Leverkusen; Germany; 100 μg/gm body weight)-Rompun (CP-Pharma; Burgdorf; Germany; 5 μg/gm body weight). Each mouse received an infection dose of 2 × 10^3 ^foci forming units (FFU; 37) of either of the two virus strains intra-nasally in a total volume of 20 μl sterile phosphate buffered saline (1× PBS; Invitrogen; Darmstadt, Germany). Weight loss and survival of infected mice was followed over a period of 14 days. In addition to mice that were found dead, mice with a weight loss of more than 30% of the starting bodyweight were euthanized and considered dead.

### Detection and inhibition of serine protease activities in homogenates of lungs from infected mice

Lungs of control and infected mice were homogenized in phosphate buffered saline 1× PBS containing 0.1% BSA using the PolyTron 2100 homogenizer (KINEMATICA; Littau/Lucerne, Switzerland). Debris was removed by centrifugation for 10 min at 6000 g. The samples were aliquoted and stored at -70°C till being used. Trypsin-like serine protease activities were quantified in aliquots from lung homogenates form both mice strains at various time points post infection (p.i.) starting from day 1 as previously reported [62] using the specific substrate Bz-Val-Gly-Arg-*p*-NA (Bachem; Bubendorf, Switzerland). This substrate contains an alkaline amino acid (arginine) in the P1 site upstream the *p*-NA group that favors proteolysis by trypsin like serine proteases at alkaline pH [9,10] leading to release of yellow coloured *p*-Nitroaniline which can be recorded by measuring the absorbance at λmax 405 nm using a micro-well plate reader (TECAN-SUNRISE, Austria). The intensity of the yellow colour is directly proportional to the enzyme activity. To further confirm the nature of the observed serine protease activities, inhibition assays were carried out using the specific inhibitors 4-(2-aminoethyl)-benzolsulfonylfluorid-hydrochlorid (AEBSF-HCl; AppliChem, Darmstadt, Germany) and *p*-aminobenzidine-HCl (*p*AB; Bachem; Bubendorf, Switzerland) as previously described [62].

The molecular weights of the serine proteases in the lung homogenates were characterized by electrophoresis on SDS-polyacrylamide gels (SDS-PAGE) copolymerized with gelatin [63], a technique known as zymography using a Mini-Protean II electrophoresis chamber (Bio-Rad Laboratories; Muenchen, Germany). After electrophoresis, proteins were allowed to re-nature by removing the SDS. This was accomplished by incubating the gel in 2.5% triton-X-100 in water with gentle shaking for 30 min and with one change at room temperature. The gel was then incubated overnight at 37°C with gentle shaking in 30 mM tris-HCl, pH 9.5, containing 60 mM NaCl and 0.05% NaN3, subsequently stained with 0.5% Coomassie blue (in 10% acetic acid, 5% methanol) and de-stained using 60% methanol. Proteolytic activities were evident as unstained bands against the blue background of the gels.

### Quantification of protease transcripts in lung tissue by real time PCR

Total RNA was extracted from lungs of control and infected mice using Trizol reagent according to the manufacturer instructions (Invitrogen) and RNA concentration was quantified (NanoDrop 1000 spectrophotometer; Thermo Scientific, Fisher Scientific, Germany). A total of 1 μg RNA was used to prepare double stranded cDNA using SuperScript III reverse transcriptase (Invitrogen) in presence of oligo dT (Invitrogen). The sequences of the primers, names and accession numbers of the target serine protease genes are listed in Table 2. Relative quantification of the serine proteases transcripts was carried out in 96 well plates (Roche, Mannheim, Germany) using the SYBR Green I Master kit (Roche) according to the manufacturer instructions and the LightCycler 480 apparatus (Roche). *Actb *(*beta actin*) and *Gapdh *(glyceraldehydes-3-phosphate dehydrogenase) were used as housekeeping genes for normalization.

**Table 2:** Nucleotide sequences of the sense and anti-sense primers as well as the annealing temperatures used in the quantification of known protease genes by real-time RT-PCR

Protease	Sense primer	Anti-sense primer	Annealing (°C)
*Tmprss2*	TGACTGCTGCTCACTGCTTT	ATGGTTTGCATCTGGGAGAC	52
*Tmprss4*	AGGGGAGGATGAGGAACACT	ATCTGGACGGATCTCCACTG	52
*Tpsg1*	GGTCACACTGTCTCCCCACT	ACTTTGGCCTCCTGAAGGTT	52
*Ctsd*	TGATGGGAGCTGGTTTCAAT	TCATCAGGGCATAGGACACA	50
*Elane*	GGCTTTGACCCATCACAACT	CGGCACATGTTAGTCACCAC	52
*Ctrl*	CCCATTGCCTCAGCAACTAT	CCAGCCTGTGACATAGCAGA	52
*Mmp 1a*	CCTTCCTTTGCTGTTGCTTC	CACCTGGGCTTCTTCATAGC	52
*Mmp 1b*	GTGCTCTCCTTCCACAGAGG	ATGGGAGAGTCCAAGGGAGT	52
*Mmp 2*	GAAACCGTGGATGATGCTTT	CCATCAGCGTTCCCATACTT	50
*Mmp 8*	AACGGTCTTCAGGCTGCTTA	GGGAACATGCTTGGTATGCT	52
*Mmp 9*	CGTCGTGATCCCCACTTACT	AACACACAGGGTTTGCCTTC	52
*Gzma*	TGATGTGAAACCAGGAACCA	ATGCCTCGCAAAATACCATC	50
*Gzmb*	GACCCAGCAAGTCATCCCTA	CACACTCCCGATCCTTCTGT	54
*Gzmc*	CCAGGGGATGAGTGCTATGT	ATCCATCAGTTTGCCCGTAG	52
*Gzmg*	CATTCCCCATCCAGCTTTTA	GATCTGCGTGGTCTTGGAAT	50
*Gzmk*	CCGTGGTTTTAGGAGCACAT	CAGGGTATCAGAGGCGGTTA	52
*Actb*	GTCCCTCACCCTCCCAAAAG	GCTGCCTCAACACCTCAACCC	55
*Gapdh*	GGTGAAGGTCGGTGTGAACG	CTCGCTCCTGGAAGATGGTG	55

### Inhibition of H1N1 and H7N7 entry into cultured lung epithelia

In 6-well plates (Techno Plastic Products; Trasadingen, Switzerland), Adenocarcinomic human alveolar basal epithelial cells (A549; American Type Culture Collection, Manassas, USA) and mouse lung epithelial cells (MLE15; ATCC) were grown at a density of 10^6^cells/well in Dulbecco's Modified Eagle Medium (DMEM-GlutaMax; Invitrogen) supplemented with 10% fetal calf serum (FCS), 1% penicillin/streptomycin and 1 mM sodium pyruvate (all supplements were from Invitrogen) at 37°C in 5% CO2 till semi-confluence. Medium was removed, cells were washed twice with 1× PBS then incubated at 37°C in 5% CO_2 _with serially diluted AEBSF or *p*AB or cocktail of both in complete medium and control wells where cells were incubated with medium containing no inhibitor were included. After 1 hour (h) medium was removed, cells were washed from any remaining traces of the inhibitors then infected with either H1N1 or H7N7 influenza virus diluted in DMEM containing 0.1% BSA to a multiplicity of infection 0.01 (i.e. 10^4 ^virus FFU/10^6 ^cells). After 1 h, medium from individual wells was separately collected from individual wells, cells were washed 2× with 1× PBS and incubated overnight in complete DMEM-GlutaMax medium containing the above mentioned supplements.

On the next day medium (containing newly released viral particles) was separately collected from individual wells. Collected medium 1 h or 1 day p.i. were briefly centrifuged at 3000 g for 5 min to get rid of any cellular debris; supernatants were aspirated into individual tubes and subjected for quantification of influenza A virus NP by enzyme linked immune sorbent assay (ELISA) as a read out for the inhibition of viral entry and propagation. Extracellular free H3N8-NP was previously detected by others in the culture medium of infected MDCK cells by [64] who demonstrated that the amount of NP correlates with the production of mature virions. Thus, anti-NP-ELISA may be used to quantify virus load both in the supernatant of infected cultured cells and in the homogenates of lungs from infected mice.

For the ELISA assay [65], 100 μl of the collected supernatants were applied onto micro-titer plates (Greiner Bio-One; Frickenhausen, Germany) and plates were incubated overnight at 37°C, then washed 3× with 1× PBS containing 0.05% tween20 (PBST). Antigen-free sites were blocked to avoid non-specific binding using PBST containing 5% fetal calf serum (PBST-FCS; 200 μL/well), incubated 1 h at 37°C and washed 3× with PBS-0.05%T. Subsequently, 100 μl/well of diluted first antibody (anti-influenza NP polyclonal goat antibody from Virostat; Portland, USA, 1:500 in PBST-FCS) was added and plates were incubated at 37°C for 2 h. After 3 washes, 100 μl/well of the diluted secondary antibody (anti-goat-HRP from KPL; Gaithersburg MD, USA, 1:500 in PBST-FCS) was added and plates were incubated at 37°C for 2 h. For visualization of the antigen-antibody binding reaction, 100 μl/well of the *O*-phenylenediamine substrate (Sigma, St. Louis, Mo., USA) diluted in H_2_O_2 _containing substrate buffer (49.6 ml 0.1 M citric acid anhydrous, 50 ml 0.2 M dibasic sodium phosphate, pH 5.00) was added and plates were left for 10 minutes at room temperature until color development. Stopping solution (2 M HCl, 50 μl/well) was used to terminate the enzymatic reaction and the changes in optical densities (OD) were recorded at λmax 492 nm using a micro-well plate reader. A higher titer of the NP in the collected medium at 1 h p.i. reflects a more prominent effect of the inhibitor to block viral entry. On the other hand, a lower NP titer in the collected medium at 24 h p.i. indicated a more prominent effect of the inhibitor to block viral propagation.

### Treatment of mice with serine protease inhibitors

Two days prior to infection with H1N1 virus, individual C57BL/6J mice received intra-nasal treatment with either AEBSF (125 μg/25 μl/mouse) or cocktail serine protease inhibitor (125 μg AEBSF, 400 μg *p*AB/25 μl/mouse). The control groups each received 25 μl sterile H_2_O. Two days prior to infection with H7N7 virus, individual C57BL/6J mice were treated intra-nasally with cocktail serine protease inhibitor (125 μg AEBSF, 400 μg *p*AB/25 μl/mouse) and also a control group of mice was included as described above. On the third day, 20 min post treatment all mice including control ones were infected with either of the two influenza strains. The protease inhibitor treatment was continued for 2 days p.i. Weight loss and survival of infected mice were monitored over a period of 14 days p.i.

### Analysis of virus replication

At day 6 p.i., lungs were excised from individually treated and control mice and homogenized for quantifying viral NP by ELISA assay as described above, and relative quantification of either the HA1 or HA7 transcripts by real time RT-PCR using specific primers (Table 3) was performed as described above.

**Table 3:** Nucleotide sequences of the sense and anti-sense primers as well as the annealing temperatures used in the quantification of the viral HA1 and HA7 transcripts by real-time RT-PCR

Target	Sense primer	Anti-sense primer	Annealing (°C)
HA1	CAGATGCAGACACAATATGT	TAGTGGGGCTATTCCTTTTA	48
HA7	TCTGCCATTCCAAAACATCA	GCAGTTCCTTCTCCTTGTGC	48

### Statistical analysis

The calculations of the mean values, standard deviation and significance were performed using the nonparametric Mann Whitney U-test provided in the GraphPad InStat statistics program. All graphs including percent body weights, viral titers, enzyme activities and inhibition were plotted using the graphics program GraphPad Prism version 4.

## Competing interests

The authors declare that they have no competing interests.

## Authors' contributions

MMB together with KS proposed the work and designed the study experiments. MB carried out most of the experiments and wrote the manuscript. KS contributed to data interpretation and manuscript writing. PB contributed to virus titration, mice infection and lung homogenates preparation. All authors read and approved the manuscript in its final form.
